# Antifragile control systems in neuronal processing: a sensorimotor perspective

**DOI:** 10.1007/s00422-025-01003-7

**Published:** 2025-02-15

**Authors:** Cristian Axenie

**Affiliations:** Department of Computer Science and Center for Artificial Intelligence, Technische Hochschule Nürnberg Georg Simon Ohm, Keßlerplatz 12, 90489 Nuremberg, Germany

**Keywords:** Antifragility, Control systems, Neuronal networks, Uncertainty, Volatility

## Abstract

The stability–robustness–resilience–adaptiveness continuum in neuronal processing follows a hierarchical structure that explains interactions and information processing among the different time scales. Interestingly, using “canonical” neuronal computational circuits, such as Homeostatic Activity Regulation, Winner-Take-All, and Hebbian Temporal Correlation Learning, one can extend the behavior spectrum towards antifragility. Cast already in both probability theory and dynamical systems, antifragility can explain and define the interesting interplay among neural circuits, found, for instance, in sensorimotor control in the face of uncertainty and volatility. This perspective proposes a new framework to analyze and describe closed-loop neuronal processing using principles of antifragility, targeting sensorimotor control. Our objective is two-fold. First, we introduce antifragile control as a conceptual framework to quantify closed-loop neuronal network behaviors that gain from uncertainty and volatility. Second, we introduce neuronal network design principles, opening the path to neuromorphic implementations and transfer to technical systems.

## Preamble

Feedback control theory offers the most versatile and comprehensive set of analysis and synthesis methodologies for dynamical systems under prescribed dynamics. In technical systems, this framework is instantiated as an engineered machinery that transforms qualitative mechanistic explanations into means of attaining prescribed dynamics in the presence of *uncertainty* and *volatility*. In contrast, in biological systems, *feedback control theory* is used in a reverse engineering way by generating qualitative hypotheses and capturing mechanisms that maintain function under the impact of *nonlinear dynamics* and unforeseen *perturbations*.

### Control-theoretic neuronal processing

*Neuronal processing* offers a complex and subtle canonical system that exposes a large repertoire of dynamics. Under the impact of perturbations that can take place over *timescales* from milliseconds to months or even years, *neural networks* need to keep their *stability*. It seems paradoxical, but the only way neural networks can stay stable is if they are excitable, able to *adapt* their response (and structure) in reaction to outside stimuli, and able to respond appropriately. This perspective is also supported by Tower’s Selectively Advantageous Instability (Tower 2024) and the variational and relational stability of resilient systems of Bettinger and Friston (2023). Interestingly, the SAI work explores the minimal set of models that might define gaining from uncertainty across multiple levels of biological organization through a “selectively advantageous instability”. Here, one typically considers explicit neural signals reflecting uncertainty Schultz et al. (2008) in the closed reinforcement learning loop and the fact that the sensorimotor system itself contains components supporting perception under uncertainty, for instance through the thalamo-cortical excitability modulation Kosciessa et al. (2021). Neural networks, like other *biological systems*, exhibit a broad *spectrum of behaviors* concerning modifications at the neuron-level excitability that regulate and improve their functionality, absorbing a wide variety of molecular and cellular parameter changes while preserving their spiking and other functionality. Here, the system that is subject to control – the mechanism of spike generation – is intricately intertwined with the control system itself. This control mechanism comprises a sophisticated apparatus that encompasses rapid mechanisms of protein and ion channel self-regulation (i.e., kinetics) as well as slower mechanisms that modulate the number of pertinent membrane proteins. However, the spectrum of behaviors is typically built with a strong reference to the basic state of stability.

In the present perspective article, the notion of *neuronal system stability* is delineated as the capacity to revert to an original, steady-state, balanced configuration following the elimination of a displacing disturbance or probabilistic input. While the concept of stability may offer limited utility in the context of biological systems due to its ahistorical nature, wherein the past of the system becomes irrelevant once it reverts to its original state, the concept remains pertinent. The intricate mechanisms that underpin the preservation of biological functionality invariably entail the engagement of numerous processes within a high-dimensional parameter space. This inherent complexity renders it virtually impossible for a biological system to revert to its original state. Consequently, the term “stability” is eschewed in the context of describing the maintenance of biological structures and functions. However, the term is employed to characterize the behavior of the system.

Considering neuronal processing, we refer to the stability of a dynamical system as the tendency to return to the initial, steady-state, balanced relations between the components that operate within it (i.e., post-spike refractory period and cell re-polarization) upon the elimination of an input disturbance. This definition is aligned with the framework of Miller & Albarracin, 2022 Miller et al. (2022), where stability is seen through the lens of three nuances of resilience, namely inertia, elasticity, and plasticity. Advancing on the spectrum, we hereby define robustness as the ability of the system to endure disturbances or variations in its input. Tightly coupled, we define resilience as the ability of a system to regain its equilibrium once it experiences a variety of variations in parameters. In this case, despite these modifications, the system reacts by modifying its internal states to preserve its general function. For instance, consider the excitability of individual neurons or neural networks that can withstand alterations in ionic channel expression, frequency of stimulation, temperature, salinity, and pH. Neurons are plastic, capable of adapting their behavior when faced with a new task, i.e., input pattern change concerning their frequency-current characteristic. This describes the next behavior in the spectrum, namely *adaptation*. This behavior is defined as the set of immediate adjustments in a cell or system (i.e., a neural network) triggered by the continued presence of a stimulus.

### Antifragility and neuronal processing

Hitherto, embedded in the formalism of feedback control theory, we extend the behavioral spectrum of neural processing. We hereby propose to consider *antifragility* as a new member of the spectrum of neural processing behaviors that goes beyond *robustness*, *resilience*, and *adaptation*. We show how it leverages the multiple time scales neural processing unfolds upon to capture how single neurons and neural networks not only absorb and react to changes but gain from volatile disturbances and uncertainty. We provide a new perspective on how single-cell, within neural networks, and between neural network dynamics work in concert, in closed-loop, to build the capacity to anticipate changes in their input and, hence, gain from the inherent uncertainty within.

The term “antifragile” was coined to denote the opposite of fragile, as defined in a book of Taleb (2012) that generated significant interest in both the public and scientific domains. Although the term has a wide range of applications, it contains a precise and mathematical definition. Systems or organisms can be defined as antifragile if they derive benefit from systemic variability, volatility, randomness, or disorder. According to mathematical definitions Axenie et al. (2024), Axenie and Grossi (2023), Axenie et al. (2022), antifragility is the nonlinear convex response that a system displays in the face of volatility to a well-defined payoff function. This is predicated on the idea that antifragility is a dynamical system’s local attribute throughout a specific area of its input space. The system could become vulnerable if its trajectory goes beyond that range. Because of its nonlinear reaction, the system is not only resilient to shocks but also antifragile, meaning it can even profit from them. According to Axenie et al. (2024), antifragility exhibits across temporal scales from short local interactions (i.e., intrinsic antifragility), to short-range modulatory interactions (i.e., inherited antifragility), and up to closed-loop driving of behaviors (i.e., induced antifragility).

#### Intrinsic antifragility

Intrinsic antifragility (or fragility) is defined as the benefit (or harm) of input distribution, unevenness, volatility, or perturbations attributed to the nonlinearity of the system’s payoff function. It is important to note the close relation of this phenomenon to Jensen’s inequality that states that the expected value of a random variable is not necessarily equal to its mean value:

1 $$\begin{aligned} {\mathbb {E}}(f(x)) > f({\mathbb {E}}(x)) \end{aligned}$$

The convex shape of the function determines the inequality. Conversely, if *f*(*x*) is concave, the inequality is flipped

2 $$\begin{aligned} {\mathbb {E}}(f(x)) < f({\mathbb {E}}(x)) \end{aligned}$$

In the event that the payoff function, *f*(*x*), representing a system, is known, then the validity of the inequality can be substantiated through direct observation of *f*(*x*). The interplay between the nonlinearity of *f*(*x*) and the statistical properties of the distribution of *f*(*x*) characterize the system dynamics. This type of antifragility relies on timescale separation describing the input–output coupling. We will treat this later in the manuscript when we describe the single neuron processing.

#### Inherited antifragility

Inherited antifragility (or fragility) is defined as the quantification of the benefit (or harm) resulting from input distribution unevenness, volatility, or perturbations attributed to the system’s response to external signals. In many scenarios, a system’s payoff function may be unknown (or unmeasurable), or it may be known but external signals introduce additional nonlinearities. A connection exists between the convexity (or concavity) of a payoff function, *f*(*x*), and the statistical properties of the distribution of *f*(*x*). For instance, variation in the input distribution passing through a convex response function, *f*(*x*), results in a right-tailed outcome distribution. In contrast, a concave response function results in a left-tailed distribution. Therefore, everything fragile must be concave to harm. This type of antifragility relies on the timescale separation and redundant overcompensation, as a means to build capacity to anticipate changes. We will treat this later in the manuscript when we describe the within neuronal population processing.

#### Induced antifragility

The concept of induced/interventional antifragility (or fragility) involves the quantification of the benefit (or harm) associated with input distribution unevenness, volatility, or perturbations. These phenomena are attributed to the system’s response to closed-loop dynamics, which is driven by a combination of various measurable quantities and prescribed system trajectories. This type of antifragility relies on combining timescale separation, redundant overcompensation, and variable structure closed-loop control that imposes prescribed dynamics under uncertainty. We will treat this later in the manuscript when we describe the between neuronal population processing.

The role of this *perspective*^1^ is to encourage the community to discuss the extension of the neural processing behavior spectrum and consider antifragility a “first-class citizen” beyond what the dynamical systems framework has already postulated. The approach takes the path of exploring the current landscape of publications and computational work in the field of computational neuroscience and frames them in the antifragility framework utilizing the analysis, modelling, and design principles of the latter. This approach acts as “feedback” from the engineering world back to the science world to better understand the brain and its mechanisms as well as to facilitate the transfer of neuronal processing principles to neuromorphic systems. Feedback control theory comes as a very useful tool to support this initiative, offering both intuitive and formal support to introduce antifragility. We are taking our first steps towards what could be a unified neuronal behavior spectrum, so we will only target, from this perspective, neuronal sensorimotor control. As such a spectrum can only be partially concretely defined through the current mathematical apparatus we have available, we believe that our steps are shedding new light on how these mechanisms work in concert to describe each member of the spectrum. We build the perspective progressively, from evidence of resilience and robustness to uncertainty coding in neuronal processing up to sensorimotor models and their inherent uncertainty. We conclude by embedding sensorimotor control in the antifragile feedback control framework, and, finally, suggesting a variety of research directions stemming from this new perspective.There is added value in bridging the very strong knowledge base in neuroscience and computational neuroscience with the engineering approaches to “elicit” novel design “recipes” which will bring neural processing principles in both hardware and software neuromorphic instantiations, such as the Neuromorphic Intermediate Representations (NIR) Pedersen et al. (2023).NIR is an open-source software initiative that proposes a shared reference framework for computations in digital neuromorphic systems. The NIR delineates a set of computational and composable model primitives as dynamical systems building blocks that integrate continuous-time dynamics and discrete events – well matching spiking neuronal dynamics. The efficacy of NIR relies in its ability to reproduce spiking neural network models of varying complexity across multiple neuromorphic simulators and digital neuromorphic hardware platforms. The key advantage of NIR and the potential for instantiating the neuronal antifragile design resides in its ability to decouple the development of neuromorphic hardware and software from the model and algorithmic development. This is facilitating interoperability between platforms and increases accessibility to multiple neuromorphic technologies.

## Neural correlates and representations of uncertainty

### Volatility and its mechanistic underpinnings

From the single neuron upstream, *uncertainty* and *volatility* are explicitly encoded in various ways. Framing uncertainty as an inherent property of the environment, central in internal models of decision-making and learning the study of Payzan-LeNestour et al. (2013) used fMRI experiments to model the encoding of uncertainty in various locations in the brain. The work defined using Bayesian tools pathways that modulate nor-adrenergic representations of uncertainty (i.e., risk, estimation uncertainty, unexpected uncertainty) in value-based decision-making. Extending this direction, volatility was defined as a nonlinear combination of onset, duration and amplitude of external signal disturbance. This view stems from the excellent study on imprecise neural computation as a source of adaptive behavior in volatile environments of Findling et al. (2021). The study defined the statistical behavior in the presence of varying and fixed volatility models leveraging: 1) high-order inferences about the environmental volatility, 2) neural computations that derive posterior beliefs, and 3) computational imprecisions that scale with the magnitude of changes in internal representations. This can be better anchored by considering Polani and colleagues empowerment framework. They proposed a versatile information-theoretic metric to describe an agent’s actuation channel in the context of sensorimotor control. Empowerment unfolds as an assessment that captures the characteristics of the apparatus as long as they can be observed because of the environment’s role in connecting sensors and actuators Klyubin et al. (2005).

### Neuronal coding of uncertainty and volatility

In a broader context it has been shown that novelty and uncertainty work in concert to regulate exploration vs exploitation Cockburn et al. (2021) in neural processing of risk and ambiguity Wu et al. (2021) by either employing a prediction error of uncertainty driving sensory learning Iglesias et al. (2021) or simply the adaptive learning under expected and unexpected uncertainty Soltani and Izquierdo (2019). Interestingly, a similar scheme was found across multiple studies where neural processing and learning were modulated through uncertainty Grossman et al. (2022), distributed representations and population coding Pouget and Zemel (2007) and studies using explicit neural representations of uncertainty and ambiguity Bach et al. (2011) for multiple tasks, of which serial decision uncertainty under probabilistic representation Van Bergen and Jehee (2019) was representative for sensorimotor control. Overall, it seems that neural coding of uncertainty and probability Ma and Jazayeri (2014) plays the central role, not only in obtaining stable and robust representations of sensory and motor streams but also in driving the neural organization of uncertainty estimates Bach and Dolan (2012). Such a representation is also compatible across timescales with the SAI coined by Tower in Tower (2024). In the context of neuronal processing, the SAI is modeled by the instability of a component (i.e., neuron/neuronal network) within a replicating system, despite its potential cost, increases the system’s reproductive fitness. SAI achieves this through mechanisms including increasing system complexity, enabling adaptation to changing environments, and maintaining diversity, often involving processes such as degradation, or the disruption of static or dynamic structure.

### Neuronal processing under uncertainty

These observations pave the way to our control theoretic framework where we embed neuronal processing. Combined experimental and modelling studies, such as of Muller et al. (2019) already characterized mechanisms for the control of entropy in neural models of environmental representations with a multivariate approach where multiple temporal and spatial scales were calibrated. This perspective emerged from a previous study which only handled partially the closed-loop perspective, where the role of dopamine was discussed in modulated affordances for active inference Friston et al. (2012) or the modulation of attention under uncertainty modeled as a free-energy problem Feldman and Friston (2010), Parr and Friston (2017). The rather vast landscape of perspectives and models in the literature makes it hard to embed all research paths in our framework but we revamp those concepts and elements which motivate our endeavour and the need to assess the varieties of uncertainty in decision-making Bland and Schaefer (2012), the impact uncertainty has upon cognitive control Mushtaq et al. (2011), and, of course, the uncertainty types and representations mediating attention, learning, and decision-making Monosov (2020).

### Closed-loop regulation of neuronal sensorimotor processing

From a closed-loop system perspective, the rather new approaches to reward-based reinforcement learning and gaining under uncertainty and volatility pave the way for extending the stable-robust-resilient-adaptive behavior spectrum. Well-known is the fact that reinforcement learning is gaining while finding optimal balance exploitation vs exploration, but this takes another dimension when uncertainty is represented under neuromodulation and attention Angela and Dayan (2005). In this context, addressing the computational basis for the representation of uncertainty by the brain, and its consequences for epistemic behavior,  Parr and Friston (2017) developed a generative Bayesian model coded for uncertainty beliefs despite noisy sensory data and volatile environments. These approaches are supported by the SAI principle in Tower (2024).

In light of the neuronal correlates of sensorimotor processing, a multitude of physiological and modelling endeavours have been undertaken with the objective of elucidating the behavioral spectrum of neuronal processing in sensorimotor convergence areas. In this context, the work in Clower et al. (2001) discussed the role of the inferior parietal lobule in sensory processing and sensorimotor integration. The findings indicate that the inferior parietal lobule is implicated in a range of functions, including the control of eye movements and attention, the mapping of extra-personal space, and the regulation of sub-cortical feedback loops from the superior colliculus, hippocampus, and cerebellum. Formulating sensorimotor integration as a closed-loop feedback system between cortical and sub-cortical structures, the superior colliculus seems to capture interesting uncertainty representations when considering visuomotor tasks. For instance, the experiments that revealed the intralaminar expectation coding during foveation Arnts et al. (2023) emphasize the interaction between intralaminar thalamic nuclei and foveation, “even though the target of the next saccade may not yet be selected, each [sensorimotor] map contains information about the probability of a visual location being next foveated” Krauzlis et al. (2013). This highlights the importance of expectation and uncertainty coding in visual processing. The ability of the brain to form expectations based on previous experiences enhances the efficiency of visual perception Summerfield and De Lange (2014), particularly at the point of fixation where “deep superior colliculus neurons use their sensory inputs to compute the probability that a target is present” Anastasio et al. (2000). This dynamic interplay is crucial for understanding how the brain processes and responds to visual stimuli in the environment Wang and Bovik (2001). Finally, as Jure (2019) and Merker (2013) suggested “due to its strategic location as a first line visual and multisensory structure to receive environmental input and its multiple direct connections from the cortex, the superior colliculus is in the interphase of several complex processes, (one of these being) bottom-up and top-down attentional interplay”. This is further corroborated by the findings of  Massot et al. (2019) in their study, which demonstrated that the superior colliculus maintains a fundamental spatiotemporal organization of neuronal populations’ activity, underlying sensorimotor transformation for feedback control. Gonzales and colleagues proposed a novel dimension of the sensorimotor alignment process in their analysis of the dynamic aspects of the superior colliculus role González-Rueda et al. (2024). They extended the alignment of the sensorimotor map, which does not only obey static rules but also kinetic domain sensorimotor convergence and its role in guiding goal-directed behaviors. This principle was subsequently modeled through a computational model of sensor fusion by  Bauer et al. (2012). Here, they proposed an algorithm that learned sensors’ reliabilities for different points in space and used their associations and reliabilities to perform fusion.

### The interplay between feed-back and feed-forward processing

Sensorimotor processing in the neocortex is contingent upon the transmission of both feed-forward and feed-back information between cortical areas Fişek et al. (2023), Siu et al. (2021), Kafaligonul et al. (2015). Feed-forward connections are ascending projections that carry information from lower to higher levels of the cortical hierarchy. Typically, the physiological response properties of neurons in a certain neuronal network are mainly inherited from feed-forward inputs, for instance in the visual cortex Briggs (2020). They are generally fast and have high efficacy Fişek et al. (2023).Feed-back connections are descending projections that carry information from higher to lower levels of the cortical hierarchy. Feedback connections are often more numerous than feed-forward connections but are weaker and slower Siu et al. (2021), Kafaligonul et al. (2015). They are considered to be modulatory. One of the roles of feed-back is to provide contextual information to lower levels, for instance facilitating perceptual functions such as contour integration and figure-ground segmentation Fişek et al. (2023). Cortical neural networks use feed-forward connections to transmit sensory information and feed-back connections to modulate and contextualize it, enabling complex sensorimotor processing. Finally, it is possible to expand the concepts of feedback control, which are now utilized to describe sensorimotor action and lay the groundwork for comprehending cognition. Specifically, the work of Pezzulo and colleagues characterizes behavior as competing and selective parallel processes among possible action possibilities (or “affordances”) represented at many abstraction levels. Expectations of their immediate consequences and rewards, as well as expectations of what additional affordances they will make accessible, skew adaptive selection among the affordances that are already available. This was further extended in Tani and Butz’s hierarchical structure, as a geometric compositional structure to describe sensorimotor processing Sugita et al. (2011).

## Robustness and resilience in neuronal processing

### Neural network behaviors spectrum

Neuronal networks need to be stable to keep track of the relationships between the different sensory and motor streams they are influenced by and their internal states. Paradoxically, such systems can only remain stable, from a dynamical systems perspective, if they are excitable, able to adapt their behavior in reaction to outside stimuli, and able to withstand those changes Cannon (1929). At the same time, neural networks are flexible, so in a way, they are seeking a balance between resource allocation for input representation, processing stochasticity, and the advantageous stability of the internal dynamics Musslick et al. (2019), Miller et al. (2022), Bettinger and Friston (2023), Tower (2024); actually, a network’s ability to accommodate slight variations in its parameters, operating variables, and state variables determines how stable it is overall Holling (1973). Finally, a neuronal network is plastic and able to adapt its behavior in response to new input configurations, tasks, and noise patterns within its components and driving variables Braun (2015).

Our goal is to shed new light and extend the stability-robustness-resilience-adaptation spectrum of neuronal processing by virtue of a novel feedback control-theoretic framework, namely antifragile feedback control systems. We build upon both theoretical dynamical systems analysis Krakovská et al. (2021) as well as biophysical evidence and modelling of single neurons Marom and Marder (2023) and neuronal networks Debanne et al. (2019) and propose a spectrum of behaviors where robustness, resilience, and adaptation are members of a broader *stability-antifragility continuum*. Such a behavioral change is supported by the anatomy, function, and structure of the underlying networks in the nervous system. As Harris and Marder suggest in Harris-Warrick et al. (1991), neuronal networks provide a physical substrate on which a vast array of modulatory inputs can operate. These inputs enable the networks to generate a multitude of output variations under diverse conditions, thereby encompassing a vast range of behaviors. This behavioral spectrum is determined by the inter-connectivity of patterns within neural networks which subsequently drives and interacts with patterns of behavior. More precisely, one needs to consider here the structure-function interplay, the time scales separation of neuronal function, and the mixed feed-back and feed-forward pathways in neuronal processing Kafaligonul et al. (2015), Briggs (2020). Such connectivity can potentially form a substrate to support both predictive and cooperative contextual interactions Fişek et al. (2023). This is supported, for instance, in the primate visual cortex neuronal processing where feed-back can rapidly and selectively influence the activity of incoming feed-forward signals Siu et al. (2021). This encompasses the mutual linkage between perception and action, as well as the influence of brain-environment interactions on cognition Bassett and Sporns (2017).

### A new member in the spectrum

As coined in the work of Taleb (2012), Taleb and Douady (2013), a dynamical system’s propensity to benefit from *unpredictability, volatility, and uncertainty* in contrast to what fragility would incur is defined as *antifragility*. The response of an antifragile dynamical system to perturbations is *beyond robust and resilient*, to the point where *stressors* can improve the system’s stability and response by contributing a significant *anticipation component*. Section 1.2 provides already some initial mathematical insights on how antifragility can be mapped onto neuronal processing. In the following, we consolidate and frame the perspective in the body of literature relevant to the behaviors spectrum.

But, in order to introduce antifragility’s role on the neuronal behavior spectrum, in the manuscript, stability refers to the ability of neural systems, (i.e., single neurons or neural networks), to return to a steady-state condition, when a volatile disturbance has ceased to act and a balance between the forces acting in the system has been restored. Robustness in this context refers to internal mechanisms that allow the neuronal system to accommodate perturbations or changes in input. Resilience is here used to characterize a neuronal system’s ability to recover from a variety of parameter changes. Finally, short-term or long-term alterations in a neuron or neuronal network process that take place while a stimulus is present are referred to as adaptation. The definitions are supported by experimental studies. For instance, Xu and colleagues 2017 Xu and Barak (2017) used inference techniques to reveal the dynamic regime in which neurons operate: a marginally stable one. They show that large oscillations in excitability are a hallmark of this regime, which also preserves great sensitivity to changes in external stimuli. This dynamic regime was captured by a model that has a dynamic recovery timeframe that interacts with excitability and accurately predicts the response of the neurons. The finding is supported by Rinberg and colleagues 2013 Rinberg et al. (2013). They show that the stability of neuronal oscillators by realistically perturbing them, especially to the point of failure, can help reveal the salient features that contribute to their robustness.

Neuronal robustness and resilience unfold across scales  Marom and Marder (2023), as seen through the lens of feedback control systems or the nature of the learning rules shared by intrinsic and synaptic plasticity and the impact of intrinsic plasticity on temporal processing Debanne et al. (2019). In a very nice mechanistic build-up, the authors introduce a behavior spectrum, from stability to adaptation through robustness and resilience. This is the starting point of our creative exercise to extend the behavior range with a new member, namely antifragility. This initiative is not only theoretical; rather, it builds upon previous research on the underlying mechanisms of robustness and resilience in neural systems. The current stability-resilience spectrum proposes that each behavior of single neurons and neural networks can seamlessly describe the transition between multiple mechanisms to sustain function in the face of disruptions occurring on timelines spanning multiple orders of magnitude due to the interleaving of distinct timescales, as suggested by the excellent framework of the comprehensive theory of adaptive variation Meyers and Bull (2002).

### Neuronal correlates of antifragility

Mathematically, antifragility can be defined as a nonlinear convex response to a well-defined payoff function exhibited by a system in the context of volatility. This definition assumes that antifragility is a local property of a dynamical system within a defined region of the system’s input space. Section 1.2 provides already the needed definitions to support the reader across the concepts of applying antifragility to neuronal processing. In the neuronal context, Calaim and colleagues gave a novel geometrical interpretation of the dynamics and function of spiking networks Calaim et al. (2022). In a systematic and thorough study of circuit-level robustness against parameter disturbances, neuronal death, and spurious noise, they have shown that neurons’ sub-threshold voltages are confined to a convex region in a voltage subspace.

To extend the spectrum, we build on the key insights from the dynamical systems analysis, as formalized in both Krakovská et al. (2021), Meyer (2015). We therein consider that: 1) resilience to recurrent state variable alterations correlates with resilience to changes in parameters due to the critical slowing down phenomenon (i.e., homeostatic activity regulation (HAR) in single neurons); 2) neuronal networks provide immunity to state variable fluctuations that may be different from those that promote recovery from them (i.e., competition and cooperation in the population of neurons encoding single sensory and motor streams through Winner-Take-All (WTA)); and 3) recovery rates matter for biological resilience (i.e., temporal correlation learning through Hebbian Learning (HL) variations).

#### Insights into neuronal intrinsic antifragility

Conceptually linked to the realm of physiological regulation based on the free energy principle and self-organized criticality Bettinger et al. introduced the concept of “variational and relational stability” in Bettinger and Friston (2023). They generalised the homeostasis feedback loop to a larger category of variational systems in biology, engineering and physics. The conceptual framework shares some traits with the antifragility definition and its instantiation in neuronal processing. Probing deeper in the realm of neuronal network stability trademarks, we consider stability in the presence of rewiring. In this context, the model of Fauth et al. (2015) illustrates how storing information in the collective dynamics of multiple synapses can resolve the contradiction between memory duration and structural/synaptic volatility. This behavior was achieved leveraging bi-stability in the number of synapses, hence supporting fluctuations in the number of synapses and leading to the additional effect of learning being orders of magnitude faster than forgetting. Such a time-scale separation of dynamics, the ground concept of intrinsic antifragility, is further supported by the study of Acker et al. (2019). Here, the research of Acker et al. suggests that synapses turn over rapidly under Hebbian plasticity, a fact that can determine memory preservation in computational models of hippocampal and cortical networks despite turnover of all synapses. Our results suggest that memory can be stored in the correlation structure of a network undergoing rapid synaptic remodeling. But the question is: how can we embed strategies and recovery mechanisms in a mathematical framework for resilience dynamics under uncertainty while still ensuring robust stability? Here we want to distinguish between robust stability and robust performance. Robust stability refers to the property of a control system that is stable (qualitatively) over changes in internal dynamics and perturbations, whereas robust performance refers to (quantitative) performance criteria being satisfied over changes in internal dynamics and perturbations Su and Samad (1997).In a very interesting study, Lara (2018) provided a definition of resilience as a form of controllability for whole random processes (regimes), whereas the state values must belong to an acceptable subset of the state set. To achieve this behavior, a mix of positive and negative feedback loops is needed to sustain this internal control signal propagation under both functional and structural changes Hebbar et al. (2022).

#### Insights into neuronal inherited antifragility

In our endeavour, the dynamical analysis of neuronal processing systems needs to be complemented by formal measures of each behavior in the stability-resilience spectrum. In this respect, the study of Bramson (2010) demonstrated that systems exhibiting feedback, nonlinearity, heterogeneity, and path dependencies need to be captured in a unified mathematical object. The study considered the Markov model framework provided above to establish formal definitions of several concepts in the dynamical systems behavior spectrum: robust, reliable, sustainable, resilient, recoverable, stable, and static, as well as their counterparts: susceptible, vulnerable, and fragile. Interestingly, recent randomly connected excitatory and inhibitory models of populations of spiking neurons illuminate the capacity of a population of neurons to respond to arbitrary variations in input stimuli, the dependence of population dynamics on the type of noise, and the influence of recurrent connections on dynamics Brunel (2000). The significance of inhibitory feedback for generating irregularity in single-cell behavior is accentuated, especially in the context of contributing to the maintenance of network states as attractors of the system. This study is further supported by the study of Denève and Machens (2016) which provided evidence that cortical inhibition offers the ability to maintain a balanced equilibrium between excitation and inhibition across time scales regardless of whether they are triggered by external stimuli or spontaneous fluctuations. In the context of induced antifragility, capacity building and time scale separation, the study, published by Timme et al. (2006), investigated the dynamics of synchronization in networks of neural oscillators with complex connections. Through their model, the researchers observed that topology-stable synchrony is common to a class of neural oscillators with a speed limit to synchronization controlled by the number of other oscillators it receives input from. The study of Luboeinski et al. (2023) proposed a model to stabilize recurrent networks by increasing the average self-coupling strength of the units of a network. For both topologies, the authors found a sharp transition from instability to asymptotic stability, which supports the characteristics of inherited antifragility. This phenomenon is further accentuated by the observation that the average self-coupling strength required for stabilization increases at a rate that is much slower than the system’s size, indicating that it gradually builds capacity to attain stability through incremental increases in unit self-regulation. This versatile framework demonstrates again the need to extend the behavior spectrum in the realm of dynamical systems with prescribed dynamics. Yet, the treatment in the study was oriented toward graph theory and statistics, whereas the practical closed-loop dynamics were not central. This motivates our perspective to stand out as an endeavour towards feedback control systems to describe antifragility.

#### Insights into neuronal induced antifragility

From a quantitative angle, there is research on wrapping probabilistic techniques for assessing the resilience of complex dynamical systems in feedback control loops. As emphasized in the study of Balchanos (2012), a feedback controller is responsible for system performance recovery through the application of different reconfiguration strategies and strategic activation of necessary redundancy. Hence, uncertainty and volatility effects on a system’s operation are captured by disturbance factors. These observations are immediately valid in the biological systems realm. In this direction, the study of Arnoldi et al. (2016) on resilience, reactivity, and variability redefines the spectrum of stable, robust, resilient, and adaptive behaviors within the framework of geometric eigenvector-based metrics. Parameterized using the eigenvalues distance from equilibrium points, these metrics capture time-scale separation and order reduction through eigenvector motion parameters.

Using the same framing of robustness in the geometrical framework of response shape in flow networks, the work of Ay and Krakauer (2007) introduces structural robustness as the core of capturing the causal contribution of each system component to the network’s robustness. This perspective is amenable to neuronal processing as functional redundancy plays a fundamental role in the robustness and resilience of the system’s response and, as we will see later, in its antifragility. Finally, the comprehensive work on resilience in dynamical systems by Krakovská et al. (2021), compiles a formal set of metrics and analysis mechanisms to describe the ability of a dynamical system to absorb changes in state variables, driving variables, and parameters and still persist. The framework was formalized along a very concise set of metrics such as return time (reaching time, proportional to the reciprocal of the eigenvalue with the largest real part of the system linearization), reactivity (the maximum instantaneous rate at which an asymptotically stable linear system responds if initial conditions are away from the origin), and intrinsic stochasticity. The study of Gallinaro and Rotter (2018) proposed a model to evaluate how Hebbian plasticity can shape neuronal connectivity during development and learning, whereas homeostatic plasticity would stabilize network activity.The study considered a recurrent network of leaky integrate-and-fire neurons, in which excitatory connections are subject to a structural plasticity rule based on firing rate homeostasis. The researchers demonstrated that a subset of neurons exhibited enhanced within-group connectivity in response to stronger external stimulation, suggesting the potential for intrinsic and inherited antifragile dynamics (see Section 1.2 for definitions) as well as a favorable integration into closed-loop induced antifragile behaviors.

This section has set the stage for the “tour de force” we perform in extending the behavioral spectrum of neuronal processing towards antifragility. We now have all the core concepts and framing defined and ready to introduce the specifics of our canonical system, namely the sensorimotor system.

## Robust sensorimotor control under uncertainty

The most appropriate way to conceptualize sensorimotor control is as a highly elaborate and intricate process that involves thousands of ensembles of peripheral sensory data processed by a network of neurons, interneurons, and central nervous system regions. This interconnected structure then uses an equally sophisticated network of pathways and neural networks to stimulate the muscles and generate coordinated movements.

### Sensorimotor control models

Typically formulated in the Bayesian framework for decision-making, its core is about constructing a representation of the state of the world used subsequently to make decisions based on describing uncertainty as probability distributions. Sensorimotor processes are plagued by uncertainty, which stems from a variety of causes including sensory and motor noise, as well as environmental uncertainty Orbán and Wolpert (2011). However, it remains unclear if uncertainty is task-dependent, only at the decision-making level, or completely Bayesian, across the whole perceptual machinery Koblinger et al. (2021), Körding and Wolpert (2004).

In order to assess the empirical adequacy and explanatory power of the antifragility framework with respect to the theory of predictive coding we analyse comparatively some recent studies. Hodson and colleagues’ 2023 study on the empirical status of predictive coding and active inference in Hodson et al. (2023) shows that empirical data provides only moderate support for predictive coding. Different feedforward models (e.g., generative Bayesian models) potentially account for the majority of empirical research on active inference. This research has been focused on fitting these models to behavior to find and explain variations between individuals or groups. This perspective is supported by Friston and colleagues 2009 study on predictive coding under the free-energy principle Friston and Kiebel (2009), Parr and Friston (2017). The study built an apparatus to map complex spatio-temporal sensory trajectories to points in an abstract perceptual space accounting for a robust representation and computation. The connection to antifragility is emphasized by the capacity of generative Bayesian models to describe the underpinnings of perceptual inference and attractor dynamics to capture the temporal development of the sensorimotor maps, using “canonical” dynamics across scales, i.e., homeostasis, competition and cooperation, and closed-loop temporal learning.

### Behavior of sensorimotor control models

Biases and optimality in cognitive decision-making form a strong reference frame when considering robustness and resilience, especially when experimental data demonstrate that motor strategy selection comes close to maximizing expected gain Trommershäuser (2009). However, this is only one pathway, whereas decision-making and movement planning are better represented in statistical decision theory. Here, economic decision-making tasks generally do not optimize expected benefits and frequently underestimate the probability of rare events Trommershäuser et al. (2008).

It seems that the sensorimotor system inherently robustifies its output on the long tails Taleb (2020) by integrating sensory and motor information, each with different noise properties (i.e., reliability), in a way that minimizes the uncertainty in the overall estimate van Beers et al. (2002). Such observations are even more well captured in the framework of affordances discovery through perception and action Chavez-Garcia et al. (2016). More precisely, sensorimotor task performance is maximized by adapting the dynamics of the system under physical and computational constraints Ogawa et al. (2006).

This perspective was further extended in the very interesting study of Friston and colleagues 2012 on dopamine-modulated affordances for active inference Friston et al. (2012). They modeled the modulation of attention under uncertainty as a free-energy problem. Yet, the empirical validity of active inference theory has not been thoroughly examined, even though active inference models often explain behavioral data rather well. This would need a formal comparison to other models, such as non-Bayesian or model-free reinforcement learning models. In this context, Miller and Albarracin conceptualized and formalised a new framework for resilience and active inference in Miller et al. (2022). They described resilience as ’inertia’ when considering the ability to resist change, ’elasticity’ when considering the ability to bounce back from a perturbation, and ’plasticity’ when considering the ability to flexibly expand the repertoire of adaptive states. In antifragility, in contrast, “elasticity” is embodied by attractor dynamics and variable structure control, and “inertia” is realized through the capacity to absorb change through time-scale separation in the antifragility framework. Lastly, the redundant overcompensation component captures “plasticity” by extending the state space despite volatility and disturbances, hence enabling robust anticipation.

### Volatility in sensorimotor control models

Overall, the capacity to absorb changes in its parameters and input and recover from volatile disruptions remains in the realm of statistical optimal perception Fiser et al. (2010). Even when formulated as feedback control loops, the computational models of sensorimotor integration still exploit the closed-loop dynamics to cope with noise in the input, disturbances and (task) structure changes Ghahramani et al. (1997).

Going beyond the neuronal correlates of sensorimotor processing, the work of  Clower et al. (2001) provided valuable modelling and processing insights into the role of the inferior parietal lobule in closed-loop control of eye movements and attention, marking a clear time scale separation and the need for rapid compensatory processes carried jointly with the superior colliculus, hippocampus, and cerebellum. These insights are then extended by the study of  Massot et al. (2019) which showed that the superior colliculus maintains a distributed representation of the spatiotemporal organization of neuronal populations’ activity specific to sensorimotor transformations in feedback control. This way prescribed dynamics are induced in the local and global feedback loops through rapid compensatory processes, used for instance in attention. Considering the physics of sensorimotor convergence  González-Rueda et al. (2024) proposed a novel kinetic domain sensorimotor convergence model. The underlying dynamics alignment of the sensorimotor maps would then generate a redundant compensatory action in response to noisy or conflicting sensory or motor streams. Such a computational model was then proposed by Bauer et al. (2012) where an algorithm that learned a sensor’s reliabilities for different points in space was combined with mapping to perform sensorimotor integration.

### Sensorimotor control interactions

It seems that the stability-robustness-resilience-adaptiveness continuum in sensorimotor control also follows a hierarchical structure Nagata et al. (1994) that explains the interactions among the different time scales of sensory integration, motor plan generation, and disturbance compensation, overall under a clear impact of coordinate transformation uncertainty Schlicht and Schrater (2007).

Interestingly, in the realm of predictive coding, the evidence is taken as the (precision-weighted) prediction error, similar to what is in antifragility played by the role of the shape of the payoff response function. Its convex shape captures the gain or loss in the face of disturbances. But, as Friston suggested in his intriguing 2018 perspective in Friston (2018), the duality of weighted prediction error and variational free energy accounts for the transition from Taleb’s antifragility as static probability distribution long-tails to the dynamic of temporal evolution of closed-loop compensatory processes. This view is then further developed by Bettinger and Friston 2023 in Bettinger and Friston (2023) who unfolded “stability through change” that includes a “return to stability” in a variational model of homeostasis. This perspective materialized in the study of Parr and colleagues 2017 in Parr and Friston (2017), where a generative Bayesian model coded for uncertainty beliefs despite noisy sensory data and volatile environments.

Multiple explanations and models have been used to capture aspects of the role of uncertainty in neural coding and computation underlying sensorimotor control Knill and Pouget (2004) where the Bayesian approaches to sensory integration Berniker and Kording (2011) seem to dominate. Yet, none of the approaches captures the consequences of fat tails Taleb (2020), statistical moments and volatility, and the geometry of the system’s response over time Taleb and Douady (2013) while learning under uncertainty Topel et al. (2023).

## Antifragility in sensorimotor processing

Antifragility was introduced as a very versatile and powerful framework to describe a system’s behavior in the face of randomness, uncertainty, and volatility by Taleb (2012), Taleb and Douady (2013). In its initial form, the mathematical treatment was centred on the probabilistic aspects of a system’s behaviors under uncertainty and volatility while describing a new fragile–antifragile spectrum of responses. At this point, we evaluate how the initial mathematical apparatus describing the antifragility principles can be applied to the realm of dynamical systems, such as neuronal networks, by probing the importance of the time component across changes in structure, function, and input. This is a crucial ingredient that allows us to define, measure, and integrate antifragility principles in control theory. This interdisciplinary research is currently carried out by the Applied Antifragility Group.

For an introduction to applied antifragility, we invite the reader to consult the work in Axenie et al. (2024), where we classify antifragility across scales: from intrinsic antifragility (i.e., describing the dynamical system’s intrinsic temporal dynamics), to inherited antifragility (i.e., determined by the system’s local interactions with other systems), and up to induced antifragility (i.e., prescribed dynamics in a closed-loop feedback control paradigm). The main ingredients of the antifragile control theory were refined across multiple instantiations Axenie and Saveriano (2023), Axenie and Grossi (2023), Axenie et al. (2022) and comprise:

- time-scale separation
- redundant overcompensation
- variable structure and attractor dynamics

Interestingly, antifragility principles found applicability even beyond our control theoretic formulation towards antifragility based on constraint-satisfaction in networks Pineda et al. (2019), robustness and fragility based on feedback dynamics Kwon and Cho (2008), antifragility in systems of systems Johnson and Gheorghe (2013), and antifragility criteria for modelling dynamical systems de Bruijn et al. (2020). This broad landscape consolidates our perspective and frames it better in the dynamical systems domain.

To facilitate the conceptual work we develop from this perspective, we anchor the explanations in a limited but representative set of neural sensorimotor control models. Initial studies framing sensorimotor neuronal processing in control theory covered classical aspects of dynamical systems controllability and stabilization Levin and Narendra (1993), observability and identification Levin and Narendra (1996), and closed-loop feedback control Narendra (1996).

Developing a computation model of inverse models building for arbitrary sensory and motor streams Hanuschkin and colleagues 2013 in Hanuschkin et al. (2013), demonstrated that Hebbian learning can be used to learn inverse models used for compensatory processes when no heterosynaptic competitive terms are used in the learning rule. This interesting result is further extended by the very insightful study of Zenke and colleagues 2017 in Zenke et al. (2017), in which the closed-loop feedback stability of the Hebbian plasticity rules was achieved through homeostatic rapid compensatory processes. This strongly motivates the antifragility theory where the “ingredients” of the temporal paradox of Zenke and colleagues 2017 are cleverly used in concert to leverage neuronal processing. Finally, in a relatively exhaustive dynamical systems treatment of Hebbian learning in recurrent neuronal networks, Siri and colleagues 2008 in Siri et al. (2008), used the apparatus of the dynamical system to shape a complete behavior analysis of Hebbian learning in a closed loop using Lyapunov exponents and the shape of the closed-loop system Jacobian curvature shape. By analysing the “graph of influences” they analysed and discussed the interplay of positive and negative feedback loops and their effect on a network’s behavior when facing disturbances.

In our current perspective, we analyse the characteristics of representative sensorimotor models through the lens of antifragility and its three main ingredients. Additionally, to adapt the framework to neuronal processing, we limit ourselves to the analysis of only three main dynamical (computational) elements: HAR, WTA, and HL, respectively. As example systems, we consider the unsupervised learning of sensorimotor relations network of Cook et al. (2010), the self-organising sigma-pi sensorimotor network of Weber and Wermter (2007), the interacting basis function network of Firouzi et al. (2014), and the sensorimotor correlation learning network of Axenie et al. (2016). Our choice of models and the computational mechanisms is motivated by multiple recent computational and experimental studies emphasizing the major role of the “canonical” computational mechanisms (i.e., HAR, WTA, HL) in neuronal processing behaviors. The most relevant studies for our current focus of describing neuronal processing within the antifragility framework are targeting transferring these principles into closed-loop technical systems. These include the computational studies of Cook et al. (2010) and Firouzi et al. (2014) and the robotic studies of Weber and Wermter (2007) and Axenie et al. (2016). In this context, the very interesting study by Tower (2024) introduces the concept of SAI of multi-component systems, such as the cellular systems. Interestingly, his framework revolves around a set of models describing the “potential cost to the system of creating unstable components and, in some cases, the [cost] of actively degrading them”. This perspective is also captured by the antifragility framework of neural processing. Considering the wide range of neuronal spiking patterns, their dynamics (i.e., neuronal activity instability or oscillations at the HAR level) contribute to the pattern of interaction (i.e., modulation by inhibition, excitation degenerating into instability, through WTA and HL) with other cells within or between neuronal populations. At the same time, this interaction pattern determines a source of stress for each cell, the processing of which is a superposition of sensory projections and local interactions (e.g., HAR + WTA + HL). Tower defines instability as “the relative propensity of a system component to undergo physical disintegration of its structure, either spontaneously or through the action of a degradative agent”. The theory of antifragility goes beyond this formulation, proposing also mechanisms to benefit from the “degradative actions” by building (anticipatory) capacity to redundantly overestimate the spontaneous effects of the structure or external actions. These actions in the antifragility theory are synonymous with SAI’s elimination of damage (i.e., degradation with benefits in addition to the release of energy) and adaptation to environmental changes by pursuing criticality. These aspects define the unifying nature of the two frameworks and the potential they have to formally capture the broader behavioral spectrum of neuronal cell processing. Finally, similar to antifragility theory, SAI operates at multiple levels of biological organization, covering intrinsic component dynamics, short-range component-level interaction dynamics, and long-range system-level interactions.

## Antifragility levels in neural processing

Our perspective unfolds, in the current section, a creative exercise that embeds sensorimotor control mechanisms in the antifragility framework. Considering a selection of relevant models and the framework set up by the perspective up to this point, we provide insights on antifragile theory-based design in Section 6, where we focus on practical aspects. The leveled approach to introduce antifragility takes into account that the “canonical” neuronal circuits we use operate on quite different time scales. The WTA dynamics operate on a short time scale, allowing the neuronal network to converge quickly. HAR and HL operate on a much longer time scale, averaging over a much larger sample of inputs.

Similarly, in their article on resilience and active inference, Miller and Albarracin provided a definition of resilience has a strong connection and similarity to the antifragility framework. In their construct, resilience is defined as multifaceted behavior across levels of sensorimotor processing. At the lowest level, resilience is described as ’inertia’ or the ability to resist change. Next, taking into account environmental interactions and second-order dynamics, resilience is seen as ’elasticity’, or the ability to bounce back from a perturbation. Finally, ’plasticity’, or the ability to flexibly expand the repertoire of adaptive states through ’slope chasing’ and ’structure learning’. In comparison, in the antifragility framework, ’inertia’ is instantiated by the ability to absorb change through time-scale separation, while ’elasticity’ is captured by variable structure control and attractor dynamics. Finally, “plasticity” is captured by the redundant overcompensation component, which allows for strong anticipation by expanding the state space despite perturbations and volatility. A very interesting point is that in their framework, Miller and Albarracin explain resilient systems as “slope chasers, seeking optimal ways to reduce error to be prepared for volatile adversities, but some threshold of error will be beyond what a system can consume”. This goes back to the core of the antifragility framework. Here, antifragility is characterised by a non-linear convex response (i.e. the sign of the curvature) to a well-defined payoff function that a system exhibits in the face of volatility. This assumes that antifragility is a local property of a dynamical system over a defined region of the system’s input space. Beyond this region, the system can become fragile. These aspects define the unifying nature of the two frameworks and the potential they have to formally capture the broader behavioral spectrum of neural cell processing.

Antifragility is a framework destined to unify neuronal dynamics descriptions across scales. From single-cell resilience and modulation of the function described by  Marom and Marder (2023) and by  Debanne et al. (2019) up to rapid compensatory processes under temporal correlation learning described by Zenke and colleagues 2017 in Zenke et al. (2017). Similar to Meyers and colleagues 2002 Meyers and Bull (2002), we develop a layered classification of antifragility types according to the level at which the adaptive variation occurs and proposed mechanisms underlying the variation. We define intrinsic antifragility describing neuron-level closed loop dynamics, inherited antifragility defined among spatially connected neurons, and induced antifragility to describe cross-population interactions and learning. More precisely, we argue that starting from homeostatic mechanisms that contribute to long-term maintenance of neuronal excitability by adapting the number of ion channel proteins present at the neuronal membrane one can already describe resilience. Subsequently, a series of hierarchical feedback control loops enable the excitability of single neuronal cells. They act upon underlying biophysical processes from fast, spike-shape modulating loops, to moderate kinetic-based regulation, and up to slow homeostatic regulation. This supports time-scale separation to promote the orchestration among the spatial and temporal interactions at the single neuron and between neural networks. Here, at the network level, Calaim and colleagues provide a geometrical interpretation of robustness in spiking networks Calaim et al. (2022). They demonstrated circuit-level robustness against parameter disturbances, neuronal death, and spurious noise, by showing that this behavior is achieved by driving neurons’ sub-threshold voltages to a convex region in a voltage subspace. Interestingly, the study of Calaim et al. indicates that subthreshold voltages are confined to a convex region in a lower-dimensional voltage space, which the authors refer to as a “bounding box.” This is strongly connected to the intrinsic antifragility characteristics. Here, the shape of the payoff function of the subthreshold voltages is confined to a bounded region with a certain shape. Here, we revamp the connection (see Section 1.2 for definitions) between the convexity (or concavity) of a payoff function over time, here subthreshold voltages, and the statistical properties of the distribution of the subthreshold voltages. The study evaluated network parameters such as the number of neurons, dimensionality of inputs, firing thresholds, synaptic weights, or transmission delays to modulate the bounding box. This is analogous with the variation in the input distribution passing through a convex response function that results in a right-tailed outcome distribution. In contrast, for this scenario, a concave response function results in a left-tailed distribution. Therefore, even in Calaim’s study everything fragile must be concave to harm. This is indeed then concluded by the authors that demonstrate the system’s functionality is preserved as long as perturbations do not destroy the integrity of the bounding box, in other words making it concave to harm.

### Intrinsic antifragility

Bottom-up, we argue that the implementation of the antifragile controller is performed through the HAR dynamics. This acts as a Proportional Derivative (PD) controller tuned in response to changes in behavioral states, experience, and learning Ruggiero et al. (2021). The study shows that mitochondria are key mediators of HAR. The release of synaptic vesicles and intracellular calcium concentration were provided as examples of neuronal variables that are known to be regulated by mitochondria. Using fundamental ideas from control theory, the study developed a classification scheme for potential homeostatic machinery parts that stabilize firing rates. However, the physiological variables underlying this process and their cellular underpinnings or neural network components are still not well identified. In a similar framework analysis endeavour, Bettinger and colleagues “re-interpreted allostasis” in order to emphasize a variational and relational foundation of physiological stability. Their work unfolded up to adopting “stability through change” to include a “return to stability” in an “improved” model of homeostasis upgraded to variational dynamics Bettinger and Friston (2023).

**Fig. 1:**
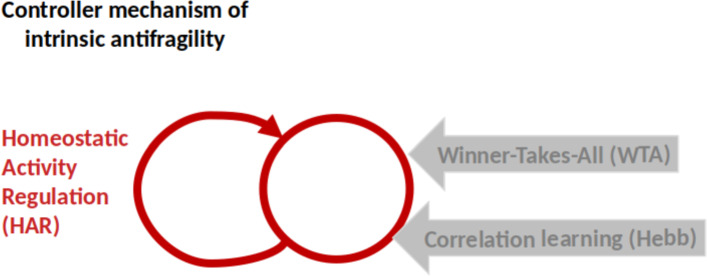
Intrinsic antifragility core mechanism based on Homeostatic Activity Regulation (HAR). The other within-population dynamics (i.e., Winner-Takes-All) and between-population (i.e., Correlation Learning) impact local dynamics, such that the neuron activation is a superposition of multiple sources with inherent own noise, distribution, and reliability properties

From the computational side, the model of Cook et al. (2010) demonstrates that HAR ensures the adaptation to local processing at the neuron level while preserving the consistent balance of *time scale separation* among WTA, HL, and HAR (see Fig. 1). This is confirmed also in the model of Axenie et al. (2016), where the HAR circuitry ensures that each neuron is active roughly a given proportion of the time, making sure that every neuron is active, and that each neuron is used in moderation.

**Fig. 2:**
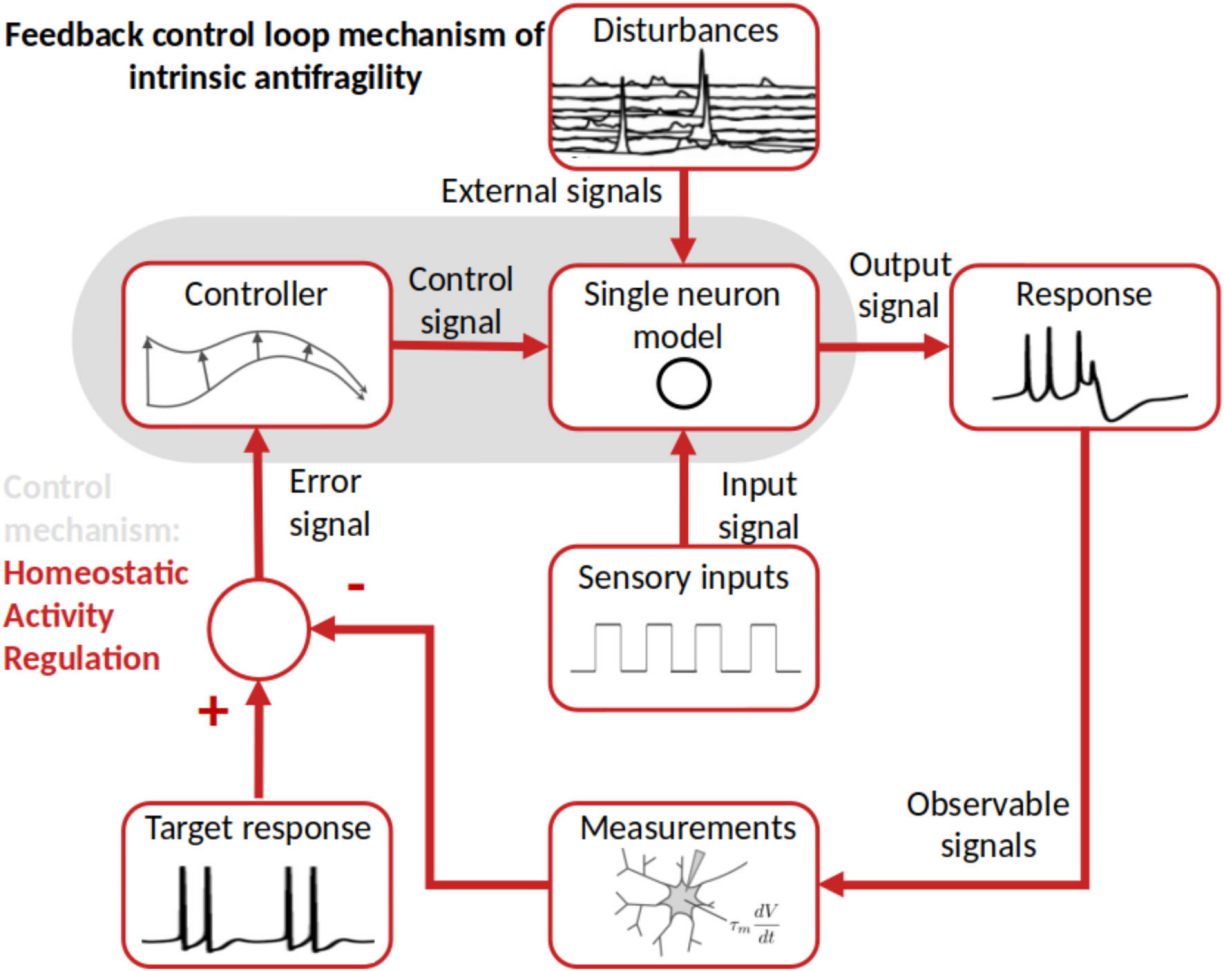
Intrinsic antifragility through Homeostatic Activity Regulation. The single-neuron model of excitability follows a prescribed response (i.e., the target response) depending on the neuron type and function, for instance, bursting or tonic spiking. Here the system to be controlled is the spike generation mechanism of the neuron model Temporal et al. (2012). At the same time each neuron, based on its localization in the cortex, receives sensory input that will code in its spiking output. This always happens in the presence of disturbances (i.e., properties of the neuron, cross-talk with other neurons, biochemical reactions in the neuron’s environment) Desai et al. (1999). The neuron’s response is a voltage that may be sensed, for instance through changes in intracellular calcium levels Liu et al. (1998), membrane voltage or pH. The fundamental component is the error signal, which represents the variation between the intended activity and the actual voltage response. The feedback controller then translates this error signal into a control signal, which controls the neuron’s expression of its channel protein membrane Schulz et al. (2006)

The antifragile feedback control loop for intrinsic antifragility builds around the basic control mechanism in Fig. 1 and describes neuron-level processing. The closed-loop feedback mechanism is here centered on the HAR dynamics implementing, together with the neuron model, the controller that compensates for disturbances and uncertainty when trying to produce the response pattern close to the prescribed target response pattern (Fig. 2).

Intrinsic antifragility captures those dynamics of the single neurons which are determined by the physical, chemical, and electrical properties of the neuron type and function. A very insightful formalism on dynamical systems behavior is offered by Krakovska and colleagues 2021 Krakovská et al. (2021). They believe that: 1) neuronal networks provide immunity to state variable fluctuations that may differ from those that promote Recovery from them (i.e., competition and cooperation in the population of neurons encoding single sensory and motor streams through Winner-Take-All (WTA)); 2) recovery rates matter for biological resilience (i.e., temporal correlation learning through Hebbian Learning (HL) variations); and 3) resilience to recurrent alterations in state variables is correlated with resilience to changes in parameters due to the critical slowing down phenomenon (i.e., homeostatic activity regulation (HAR) in single neurons). This view is then closely supported by the construct of Bettinger and Friston 2023 on physiological regulation based on the free energy principle and self-organized criticality Bettinger and Friston (2023). Here the focus changes towards, unifying, similar to the antifragility framework, the dynamics from homeostasis to allostasis, closed-loop regulation and allostatic load, tapping into redundant overcompensation, a fundamental “ingredient” of both intrinsic and inherited antifragility.

### Inherited antifragility

**Fig. 3:**
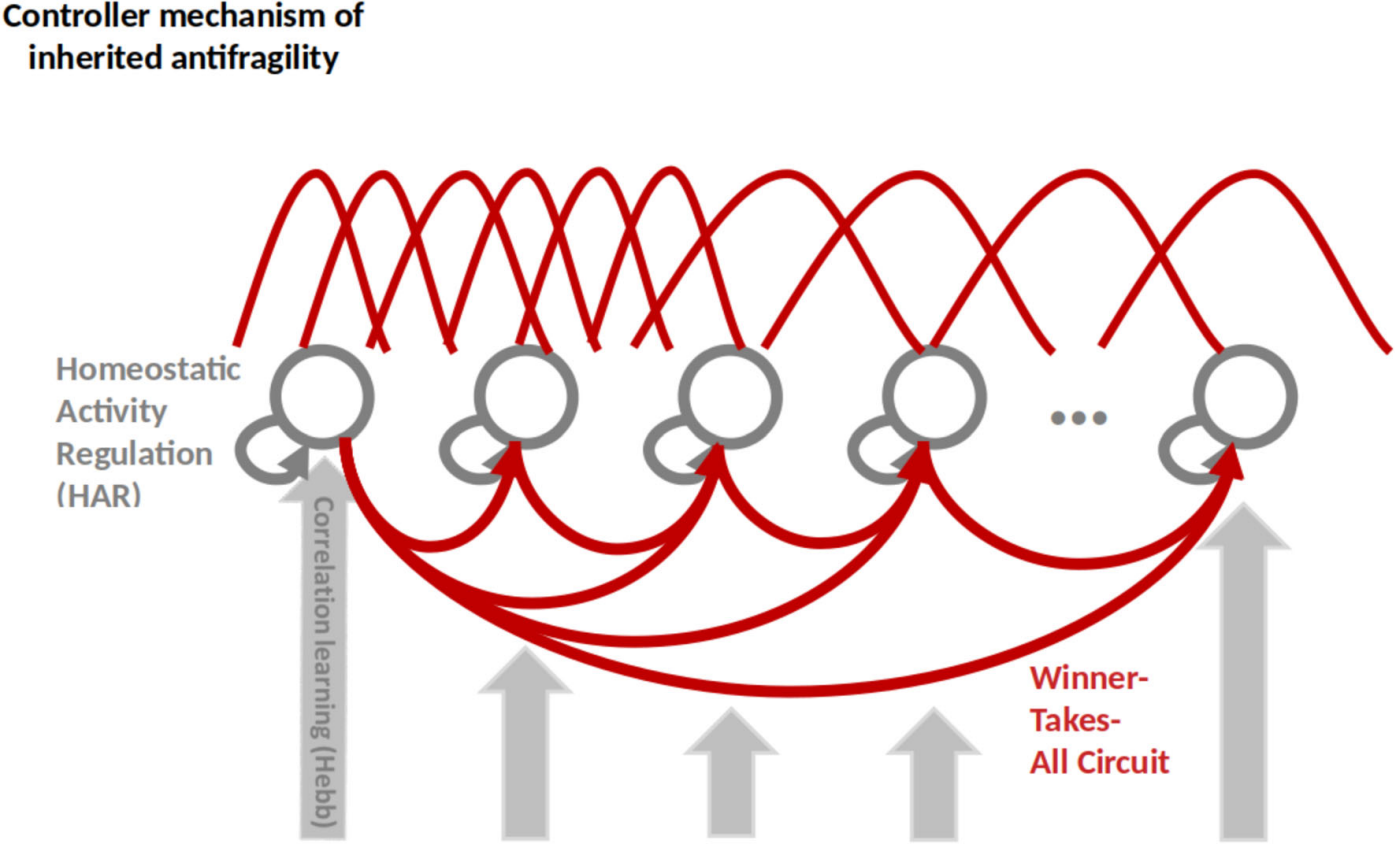
Inherited antifragility core based on Winner-Take-All within-population dynamics. Tuning curve modulation determines capacity building and sensorimotor input data distribution used in the competition and cooperation mechanisms acting between the neurons, whose activity is HAR controlled. This is also very well captured in the work of Schöner on dynamic neural fields and synergies Strub et al. (2017) used as building blocks in architectures to model sensorimotor embedding of cognitive processes. The tuning curves modulation (i.e., width and density) is coding the input space distribution. A denser input space value range is coded through multiple neurons with narrower tuning curves, whereas sparser input space value ranges through less neurons with broader tuning curves

Considering the neuronal network level, we hereby introduce inherited antifragility. At this point, neuronal population dynamics (e.g., competition and cooperation, depicted in Fig. 3) dictate the shape of the closed-loop dynamics. The superimposed effect of HAR and WTA now dictates the within-population self-organization, through time-scale harmonization and redundant overcompensation through a judicious modulation of neurons’ tuning curves.

**Fig. 4:**
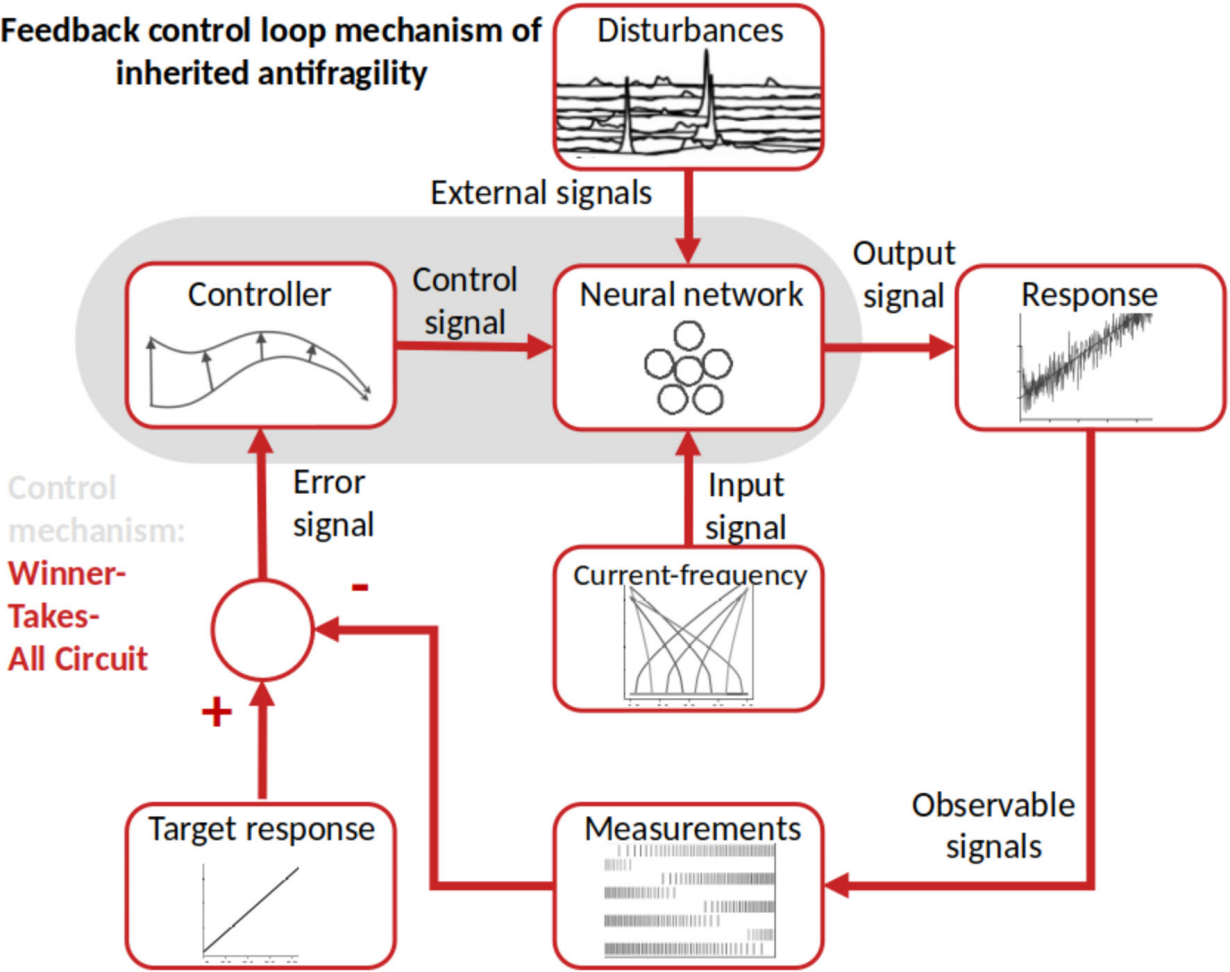
Inherited antifragility through Winner Takes All. The neural network model encodes a specific input–output function with a prescribed shape (i.e., the target function) depending on the function of the network, for instance, sensory or motor. Here the system to be controlled is the neural network spiking pattern to generate the input–output function. This always happens in the presence of disturbances (i.e., properties of the composing neurons, cross-talk with other neurons). The network’s response is a noisy version of the input–output function that may be sensed, for instance through changes in the output spiking frequency of the network’s neurons. The fundamental component is the error signal, which represents the variation between the target function and the actual function spiking response. The feedback controller then translates this error signal into a control signal, which controls the neural network’s update of synaptic connections among the neurons

In this context, the study of Kim and Lim (2022) sheds light on the dynamical origin of WTA competition within neuronal populations. Using a network of the hippocampus dentate gyrus, the study examines the dynamical origin of WTA which results in sparse activation of the granule cell clusters. Accordingly, WTA dynamics arise from a competition between the inhibitory cells’ feedback and the firing activity inside each neuronal cluster. The biophysical results are further confirmed by the computational sensorimotor control studies of Cook et al. (2010), Weber and Wermter (2007), Firouzi et al. (2014), Axenie et al. (2016), and Mirus et al. (2018). Herein, WTA circuitry is responsible for balancing time scale separation, by ensuring fast convergence but also by building capacity (i.e., redundant overcompensation) of the neuronal representation of the sensorimotor streams. Complementing slower HAR dynamics, WTA implements at the neuronal population level the antifragile controller responsible for reproducing the prescribed response under the effect of disturbances, local HAR dynamics, and the impact of the inhibitory and excitatory within-population dynamics (see Fig. 4).

But, other possible “instantiations” of competition and cooperation that might underlie the inherited antifragile behavior could be great candidates for the underlying dynamics. For instance, the study of Deco and Rolls (2005), suggested that attention could be seen as a nonlinear property that results from a top-down biasing effect that influences the competitive and cooperative interactions that work both within cortical areas and between cortical areas. Their work could support the inherited antifragility principles, especially considering the role of cooperation and competition in the dynamics of feed-back connections between cortical areas and their optimally parametrized weaker strength than the feed-forward connections in an attentional network. This provides an interesting consistency check with the time-scale separation and the redundant overcompensation built upon slower connections. These observations come close to the study of Lagzi and Rotter (2015), where for a certain range of the “within” versus “between” neuronal populations connection weights, the network activation spontaneously switches between the two sub-networks of the same type, characterized by a phase portrait with two attractors and a saddle node (i.e., a balanced excitation and inhibition in case of external drive). Finally, in a closed-loop robotic scenario, the work of Wischmann et al. (2007) proposed a combination of mechanistic and evolutionary perspectives to understand the emergence of communication among neuronal populations from an evolutionary perspective. This is inline with the antifragile design principles as described in the seminal work of Axenie et al. (2024). Inherited antifragility describes the population-level compound dynamics of time-scale harmonization and modulating action of competition and cooperation to represent the sensorimotor input signals.

### Induced antifragility

At the highest level, the interplay of both fast and slow timescales of the WTA and HAR circuits is combined with temporal correlation learning exhibited between neural populations Cook et al. (2010), Firouzi et al. (2014), Axenie et al. (2016). Temporal correlation learning rules, such as Hebbian learning, shape the dynamics and structure of neural networks, as the study of Siri et al. (2008) shows. The study provides excellent insights into effects involving a complex coupling between neuronal dynamics and synaptic graph structure that introduce both a structural and a dynamical point of view on neural network evolution.

The eligibility-weighted Hebbian learning rule proposed by Hanuschkin et al. (2013) can give rise to control-theoretic inverse models encoded in synaptic weights from sensory to motor neurons without the need for error signals or back-propagation. Superseding, these insights in the strength of Hebbian learning, the study of Zenke and colleagues 2017 in Zenke et al. (2017), provided a putative framework for rapid compensatory processes in neuronal processing. They argue that time scale separation is a requisite to stabilize Hebbian learning through the mechanism of homeostatic activity regulation and hence a modulation of plasticity. Interestingly, they provided suggestions for putative models using switch-like control signals over plasticity by gating back-propagating action potentials. Various elements that conspire to functionally shape neurobiological circuits to behave robustly or beyond through the lens of structure, function, and variability of the neurons Arle et al. (2020).

These principles are further expanded in the computational studies of Cook et al. (2010), Weber and Wermter (2007), Firouzi et al. (2014), and Axenie et al. (2016). Here, the interplay of HAR, WTA, and HL demonstrates how nonlinear sensory-motor correlations are extracted unsupervised from the noisy input streams. Additionally, efferent motor copies are used to generate plausible control inputs based solely on the sensory input and learnt sensorimotor correlations, as shown in the study of Axenie et al. (2016).

**Fig. 5:**
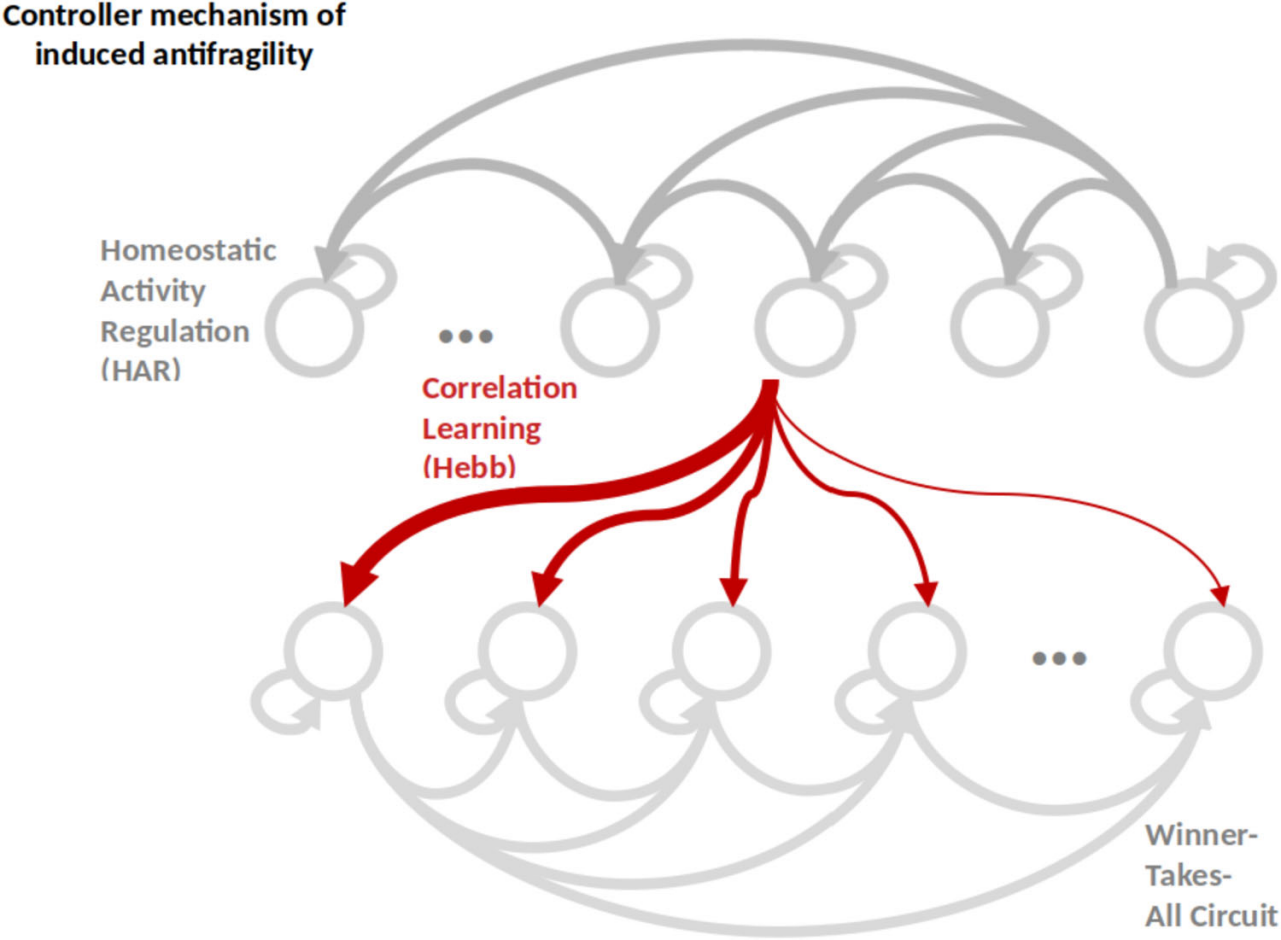
Induced antifragility core based on Hebbian Correlation Learning dynamics that capture and exploit temporal correlation of the sensorimotor input streams coded in the two interacting neuronal populations coding for sensory or motor quantities. The between population connectivity modulation (i.e., width of the line marking stronger weight) is coding the temporal correlation of the active neurons in the connected populations. A stronger correlation is coded through a thicker connection weight, whereas a weaker temporal correlation a thinner connection weight. Please note that the Hebbian connection weights are mathematically depicted as a matrix describing the all-to-all connectivity of the neurons in the coupled populations

This prescribed dynamics-guided interplay of HAR, WTA, and HL is exploited in Cook et al. (2010) to demonstrate cue integration, inference, de-noising, and decision-making in sensorimotor representations within a single neural network. This is further extended to radial basis functions in Firouzi et al. (2014), and reliability-modulated tuning curves in Axenie et al. (2016). Here, attractor dynamics compute optimal output signals that enable tracking of the prescribed dynamics between two neuronal populations based on attractor dynamics and temporal correlation learning. These computational studies were also validated in closed-loop robotic implementations in Weber and Wermter (2007) and Axenie et al. (2016), demonstrating the powerful transfer capacity of the antifragility concepts beyond analysis and modelling.

In order to evaluate the potential, the suggested “canonical” neuronal circuits (i.e., HAR, WTA, HL) have in a noisy sensorimotor processing task, we turn our attention to the closed loop neuro-robotics task in [80]. There, a robot moves in an uncluttered environment while an overhead camera tracking system keeps track of its position and orientation in the presence of different types of volatile disturbances (i.e., unknown on-/offset, amplitude, and duration). The robot is equipped with an inertial measurement unit, consisting of a 3-axis gyroscope and a 3-axis magnetometer which acts as vestibular input; wheel encoders acting as proprioceptive input; motor driver providing an efferent copy of the pulse-width modulated voltage signal sent to the 3 wheels; and a camera for visual input. This raw sensor data is fed to the multiple models (i.e., cortically-inspired neural networks Cook et al. 2010; Axenie et al. 2016; Weber and Wermter 2007, and Firouzi et al. 2014) which update their internal belief and infer an estimate of the robot’s position and orientation. To simplify the analysis, we decouple the robot kinematics and only consider a nonlinear sensorimotor mapping accounting for orientation computation from raw inertial and efferent motor copy. This accounts for learning unsupervisedly a power-law function (3rd order). The preliminary implementations of the computational studies of Cook et al. (2010) and Firouzi et al. (2014) and the robotic studies of Weber and Wermter (2007) and Axenie et al. (2016) in Table 1 are available at FauBox Uni Erlangen Repository^2^ The models in Table 1 are only example candidates. In the next steps of the exploration, we will consider also other relevant models such as: the nonlinear canonical correlation analysis neural network model of Hsieh (2000); the dynamic field theory architecture of Sandamirskaya and Schöner (2010); and the network to interpret input from a neuromorphic sensor by means of recurrently interconnected areas of Cook et al. (2011).

**Table 1:** Analysis of antifragile design principles to computational canonical circuits relevant for sensorimotor learning.

Neuronal network models antifragility elements			
Model	Time-scale separation	Redundant overcompensation	Variable structure control mechanism
Model of Firouzi et al. (2014)	Hebbian Learning, Divisive Normalization	Tuning curves modulation, Radial Basis Functions	Line Attractor
Model of Cook et al. (2010)	Homeostatic Activity Regulation, Winner-Takes-All, Hebbian Learning	Population Coding	Line Attractor
Model of Weber and Wermter (2007)	Self-Organising-Maps	Population coding	Hebbian Learning
Model of Axenie et al. (2016)	Homeostatic Activity Regulation, Winner-Takes-All, Hebbian Learning	Tuning curves modulation	Line Attractor

Compared models were used in a simple neuro-robotics sensorimotor learning task.

In this context, we postulate that antifragility is achieved through the orchestration of HAR, WTA, and HL, as shown in Fig. 5. There, time scale separation enables consistent adaptation to local changes, competition and cooperation ensure fast convergence and capacity building to anticipate changes in input data distribution, whereas correlation learning enables fast convergence towards the prescribed dynamics.

**Fig. 6:**
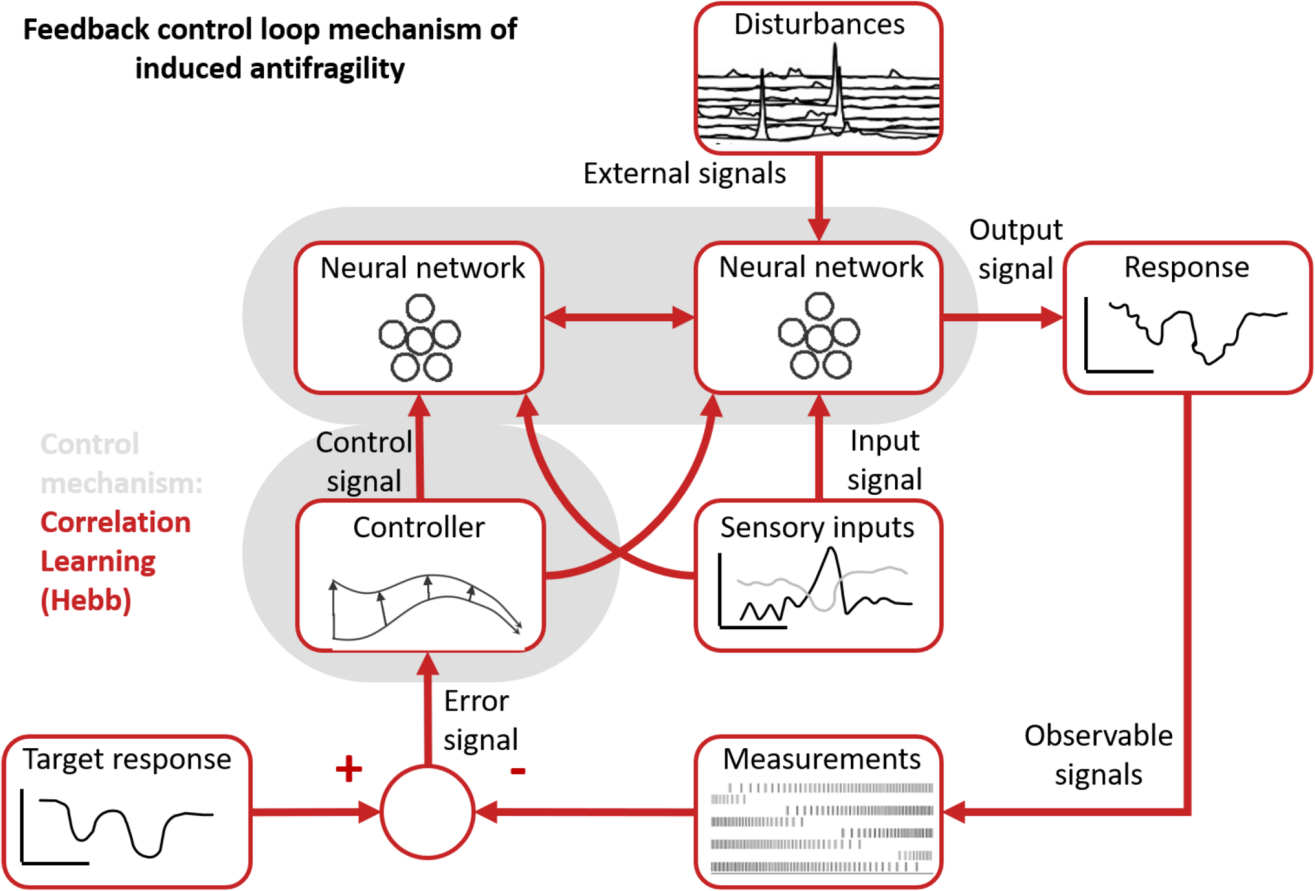
Induced antifragility through Hebbian Learning. The neural network model encodes a specific cross-sensory or sensory-motor function with a prescribed shape (i.e., the target function) depending on the function of the interacting neural networks, for instance, coding for sensory or motor quantities. Here the system to be controlled is the pair of neural networks spiking patterns to generate the joint cross-sensory or sensory-motor function. This always happens in the presence of disturbances (i.e., properties of the composing neurons, cross-talk within each neural network). Each neural network’s response is a noisy version of the input–output function that may be sensed, for instance through changes in the output spiking frequency of the network’s neurons. This is then reflected in the accuracy of the cross-sensory or sensory-motor relation maintained among the two neural networks. The fundamental component is the error signal, which represents the variation between the target function and the actual function spiking responses of the two populations concerning the joint cross-sensory or sensory-motor function. The feedback controller then translates this error signal into a control signal, which controls both the neural networks’ update of synaptic connections among the neurons

When considering network-level induced antifragility, Hanuschkin and colleagues 2013 Hanuschkin et al. (2013) show that Hebbian learning can be used to learn inverse models used for compensatory processes when no heterosynaptic competitive terms are used in the learning rule. In this context, closed-loop feedback stability of the Hebbian plasticity rules is achieved through homeostatic rapid compensatory processes, as suggested by Zenke and colleagues 2017 citezenke2017temporal. This argument strengthens the induced antifragility definition, where driving signals are used to shape the closed-loop behavior of Hebbian learning as quantified by Lyapunov exponents and the shape of the closed-loop system Jacobian curvature shape Siri et al. (2008). They examined the interaction between positive and negative feedback loops and how they affect a network’s behavior in the face of disruptions by analyzing a “graph of influences” analogue to the geometric interpretation of the payoff function curvature (Fig. 6).

In a similar initiative to conceptualise and formalise the behavioral diversity of biological systems with the ones of Miller, Albarracin and Tower, the perspective of Bettinger and Friston employs a novel physics-based mathematical apparatus to model and analyze physiological regulation. They go beyond homeostasis to define the broader class of “allostasis” dynamics, which emphasize a variational and relational foundation of physiological stability. They re-cast the Tower’s concept of selectively advantageous instability and the “chase for slopes” of Miller and Albarracin by adapting the role of allostasis to enable “stability through change”. Moreover, they integrate a “return to stability” as a means of unifying Bettinger’s “elasticity” and Tower’s “removal of damage” into a comprehensive framework. They, finally reframe homeostasis with a conceptual model of criticality that permits the advancement to variational dynamics. This extends the same adaptive valence from Miller and Albarracin’s “plasticity” and Tower’s adaptation to the environment. The concept of allostasis has emerged as a new paradigm that challenges the long-standing theory of homeostasis. Similarly, the concept of antifragility is emerging as an extension for the established concepts of robustness and resilience. A well-calibrated interplay between local and global feedback loops enables physiological regulation to be based on conservative and responsive signals from both the internal and external environments, as well as on anticipative and proactive dynamics. Antifragility can anticipate change by building capacity, and can be seen as a superset of Bettinger’s “allostasis”. It combines feedforward regulatory action, which provides stability in the face of steady disturbances, with a derivative component that responds to an anticipatory cue before a change in the environment. This concept is analogous to the “chase for slopes” concept developed by Miller and Albarracin. Time scale separation is a key enabler of biological self-organisation, playing a pivotal role in both the antifragility framework and Bettinger’s allostasis instantiations. It acts as a unifying factor between intrinsic, inherited, and induced antifragility, enabling the simultaneous “removal of damage” and variational adaptation to internal and external environmental changes. It can also be viewed as a temporal depth of self-organisation, as evidenced by the anticipatory or pre-emptive aspect of Bettinger’s allostasis. At the highest level, when we consider Miller and Albarracin’s “plasticity”, Towers’ adaptation, and Bettinger’s “allostasis”, we can see that the induced (closed-loop) antifragility offers mechanisms to enable variational dynamics instantiated through variable structure control and attractor dynamics. This main ingredient of antifragility pushes the closed-loop dynamics to minima with low curvature (i.e. basically a special property of the free energy landscape, as demonstrated by Miller and Albarracin and Bettinger and Friston alike). From selective advantageous instability to resilience as inertia, elasticity, and plasticity, and from the various instantiations of allostasis to antifragility, the conceptual landscape of neuronal processing behaviors could be described by a state of optimal anticipatory oscillation. This is hypothesized to relate to the state of criticality, as emphasized by Bettinger and Friston. Additionally, the study of Timme et al. (2006), strengthen the hypothesis that the fan-in factor of a neural oscillator can determine a stable synchronization of the network. Moreover, the study of Denève and Machens (2016) provided evidence that cortical inhibition offers the ability to maintain a balanced equilibrium between excitation and inhibition across time scales regardless of whether they are triggered by oscillatory inputs. Finally, the work of Brunel (2000), puts all the concepts together clarifying the capacity of a population of neurons to respond to arbitrary variations in input stimuli base on the statistics of the input and the feed-back connection in the network’s structure. These frameworks employ different constructs to model and analyse the brain’s dynamics away from a critical point to a “quasicritical” state, subject to constant external stimuli. This type of external drive optimises the brain’s responsiveness to stimuli, which is a key factor in how the brain processes information.

In their neuronal network-level robustness study, similar to Marom and Marder (2023), Calaim and colleagues demonstrated passive redundancy in the low-dimensional sensorimotor signals representations in spiking neurons which bind geometric insights neurons responses with the biophysical correlates. This way, the notion of antifragility can easily extend the “bounding box” geometry in a high-dimensional generalization of a network’s payoff function. Interestingly, the variable structure dynamics through attractor dynamics at the core of the antifragility framework can also compensate for the “ping-pong” effect reported by Calaim and colleagues through a judicious choice of driving signals that account for an increased “bounding box” size and ablation of strong excitatory connections. These studies are supported also by the great analysis of Debanne and colleagues 2019 which discussed the nature of the learning rules shared by intrinsic and synaptic plasticity and the impact of intrinsic plasticity on temporal processing Debanne et al. (2019).

Our perspective complements the current tendencies in neuronal control systems where the event-based control paradigm unfolds over nested timescales Sepulchre (2022). Here, positive feedback is responsible for enhancing or amplifying change, whereas negative feedback dampens and buffers changes. The resulting mixed (positive/negative) controller acts as a monotone operator, which shapes the sensitivity of the closed-loop system and its excitability. Our paradigm goes away from typical equilibrium designs and describes behaviors which go beyond robustness.

## Outlook

In our current study, we introduce the novel concept of neuronal processing antifragility in the context of sensorimotor control. In this perspective, we probe and analyze qualitatively if that time scale harmonization can drive a sensorimotor network’s evolution trajectory, through a judicious time scale separation within HAR, WTA, and HL. Having a network capable of structure variability through line attractors enables inference/learning relations among sensorimotor streams, de-noising, fusion, and decision-making. Finally, redundant overcompensation through the modulation of the closed-loop system builds capacity (i.e., redundant overcompensation) and ensures a fast reaching of the desired dynamics. The current perspective opens the path for more computational research aiming at embedding antifragility concepts in the NIR and then building interactive neuro-robotics systems as in Mirus et al. (2018). This way the effort carried out to develop a mechanistic implementation of antifragile control systems such as the one in Axenie and Saveriano (2023) would then boil down to instantiate NIR in order to leverage the advantages of neural computation. The purpose of this study is to instigate the community to consider antifragility as a novel analysis and design paradigm, shedding new light on the “canonical” neuronal computation mechanisms and their translation in practical neuromorphic systems implementations. We believe that antifragile analysis and design can provide a new fruitful research direction in both computational models and their practical instantiations in technical systems.

## Data Availability

No datasets were generated or analysed during the current study.

## Footnotes

1 Research designed and developed by the interdisciplinary Applied Antifragility Research Group.

2 https://github.com/ki-zentrum/bio-cybernetics-antifragility-codebase.
